# Some Aspects of Medical Science

**Published:** 1894-06-30

**Authors:** 


### Some Aspects of Medical Science.

We propose dealing in this, and in some other papers to follow, with a comparison between the relative senses of man and woman, with the differences existing in the acuteness and precision of the respective sexual perceptions. A vast amount of significant evidence on this most interesting subject has been brought together recently by Mr. Havelock Ellis, who bases his comparisons on an exhaustive research amongst all available material on the subject.

If we have been deluded into giving the female sensory organs a greater keenness than the male, Mr. Ellis does something to set us right in this direction. The impression—and it is a widespread one—is due, it seems, rather to a confusion of terms than to any circumstantial observations. The terms which have thus become confused—*i.e.*, those of sensibility and irritability, or affectability, possess quite opposite qualities. The first means precision and perception of stimulus, the second being the readiness of motor response to stimulus. Coleridge it was, whose keen perception pointed out one of the greatest sexual differences existing in this direction. Galton, again, who was a pioneer investigator in sexual sensory differences, remarks on the subject, "At first, owing to my confusing the quality (sensitivity) of which I am speaking, with that of nervous irritability, I fancied that women of delicate nerves who are distressed by noise, sunshine, &c., would have acute powers of discrimination. But this I found not to be the case. In morbidly sensitive persons both pain and sensation are induced by lower stimuli than in the healthy, but the number of just perceptible grades of sensation between them is not necessarily different. I found, as a rule, that men have more delicate powers of discrimination than women, and the business experience of life seems to confirm this." . . .

One of the latest authorities on the subject insists that there is considerable ground for us to consider the female sense as less keen, and Mr. Ellis and many others base their conclusions on Burdack's opinions, which are in harmony with much that is known of the sense organs of a lower range of being in animals. The antennæ of insects, the hair and eyes, are nearly always more developed in the male. Of course, in the instance of a higher order of being it must always be borne in mind that women, as Mr. Ellis points out, are habitually subject to a less thorough education, which necessarily makes them remain in a more rudimentary state.

A great deal of evidence tending in this direction has been brought to bear in the comparative sexual equality from the sensory point of view of pupils in American and other educational colleges. Again, as our authority points out, an increased keenness of tactile sensibility can be obtained through practice in lower animals. In this paper we shall devote ourselves to a study of the comparative sensibility to pain in the sexes, and decide upon a conclusion in this case, which is not, perhaps, quite in accordance with the results obtained by a study of other senses in the two sexes.

An Italian scientist who gave some years of study to the question of the relative sensitiveness to pain in men and women submitted his theories to a practical test, the medium he applied for the tests being the electric algometer. This pointed to the conclusion that there was a markedly greater sensibility to pain in man, the figures for men and women of the people being 69·23 for the former, 53·16 for the latter, the difference being less but still marked in young persons. Another measure brought to bear upon this question also by an Italian, is stated by Mr. Havelock Ellis to be in the instance of a light hammer pivoted at a point 200 m.m from its iron head, thus allowing of its falling on the tip of the forefinger of each hand, both finger and hand being supported. The minimum number of degrees through which the hammer must fall in order to cause a painful sensation was found to be surprisingly constant, and, as might be expected, it was much smaller in women. The figures for the right hand are given in men at 33·9, in women 16·6; for the left hand, men 22·7, women 14.8. As regards the left hand, Mr. Ellis points out that men and women are more nearly equal, but that there is a very considerable disproportion as regards the right hand.

Of course these results do not lead to any really definite conclusion, for it must constantly be borne in mind the immense mental part which is also brought to bear. It is a fine point, and one which has not been settled yet, the determination of where the power of courage and of endurance ends and the sensibility to pain commences. What really does seem to bear in no inconsiderable degree on the subject relates to the question of disvulnerability in the two sexes. Among the lower animals there is a high degree of disvulnerability. Among different races this appears to be everywhere well marked, according to the observations of our authority, and further, it appears, to be associated with a measurably high degree of insensibility. The negro has been proved to possess a greater reparative power after injuries than the white man. At the same time, we are reminded that among different nations the degree of bearing pain does not by any means necessarily seem to be related to the evolutionary scale of the race. The Turks show an astonishing indifference to physical suffering. A French surgeon, who has given us his opinion, declares that operations, in his experience, are borne far better by women than by men. In a total of 1,244 cases of amputations in men, he reports there were 441 deaths, *i.e.*, 35·45 per cent. In a total number of 284 cases of amputation in women there were 83 deaths. A considerable difference is here apparent, a difference possibly connected, it has been suggested, with the well-recognised resistance to death shown not only at birth by female infants, but in old age in the greater longevity of women.

And thus, having considered this subject in a general and wide sense, what are the conclusions which can be formulated as to the respective sensitiveness of man and woman to physical pain? The conclusion which Mr. Havelock Ellis arrives at is one which is in accord with the one most readily accepted, and that is, that notwithstanding their greater nervous irritability in most respects, women are better able to resist pain and discomfort than men; and after mature consideration, he dismisses most evidence as insufficiently strong to prove the diminished sensitiveness to pain in women as compared with men.

